# Efficacy of combined anti-VEGF and photodynamic therapy for bilateral diffuse uveal melanocytic proliferation: Case series

**DOI:** 10.1097/MD.0000000000027578

**Published:** 2021-10-22

**Authors:** Manabu Miyata, Sotaro Ooto, Masayuki Hata, Ayako Takahashi, Akitaka Tsujikawa

**Affiliations:** a Department of Ophthalmology and Visual Sciences, Kyoto University Graduate School of Medicine, Kyoto, Japan; b Department of Ophthalmology, Maisonneuve-Rosemont Hospital Research Centre, University of Montreal, Montreal, Canada; c Biochemistry and Molecular Medicine, Maisonneuve-Rosemont Hospital Research Centre, University of Montreal, Montreal, Canada.

**Keywords:** anti-vascular endothelial growth factor therapy, bilateral diffuse uveal melanocytic proliferation, combination therapy, photodynamic therapy

## Abstract

**Rationale:**

Bilateral diffuse uveal melanocytic proliferation (BDUMP) is an extremely rare retinal exudative disease with physical disorders and no established treatment standard. We describe treatment courses in 3 cases of BDUMP.

**Patients concerns:**

Three male patients complained active vision loss. One male patient in his 70s (patient 1) was treated with prednisolone, mesalazine, and ciclosporin for hypoplastic anemia and ulcerous colitis. One male patient in his 60s (patient 2) was on prednisolone therapy for adult Still disease. Another male patient in his 70s (patient 3) was on prednisolone therapy for polymyalgia rheumatica, giant cell arteritis, and pancreatic body tumor.

**Diagnoses:**

Retinal specialists diagnosed these patients with BDUMP based on characteristic fundus findings of multiple red patches and retinal exudate.

**Interventions:**

Two patients (patients 1 and 2) with poor response to anti-vascular endothelial growth factor (VEGF) monotherapy and/or triamcinolone acetonide sub-Tenon injection were treated with combined anti-VEGF therapy and photodynamic therapy. One patient (patient 3) was treated with 3 rounds of monthly anti-VEGF monotherapy.

**Outcomes:**

Retinal exudates were resolved in all patients. No recurrence of retinal exudates was observed for at least 10 months, 2 years, or 4 months after the therapy in patients 1, 2, and 3, respectively. However, best-corrected visual acuity of the right eye was low (20/200) compared with that of the left eye (20/22) in patient 2 despite exudate resolution, due to permanent outer retinal damage secondary to long-term retinal exudate.

**Lessons subsections:**

Combined anti-VEGF therapy and photodynamic therapy may be a feasible therapeutic option for treatment-resistant exudate in patients with BDUMP. Early diagnosis of BDUMP and prompt administration of combination therapy are crucial.

## 1 Introduction

Bilateral diffuse uveal melanocytic proliferation (BDUMP) is an extremely rare retinal disease with physical disorders. It is characterized by exudative retinal detachment and multiple round or oval, sable red patches at the level of the retinal pigment epithelium (RPE) in the posterior fundus.^[1]^ A systematic review of case reports showed that the visual disturbance progresses rapidly.^[2]^ Although several treatment methods, including periocular triamcinolone acetonide injection,^[3]^ intravitreal anti-vascular endothelial growth factor (VEGF) injection, and photodynamic monotherapy have been reported, some patients, nevertheless, experience progressive visual loss despite these treatments.^[4]^ Combined photodynamic therapy (PDT) and anti-VEGF therapy could weaken choroidal neovascularization more strongly than anti-VEGF monotherapy.^[5]^

Herein, we describe the cases of 2 patients with BDUMP who were treated with combined PDT and anti-VEGF therapy after confirmation of poor effectiveness of anti-VEGF monotherapy, and the case of 1 patient with BDUMP who was treated with anti-VEGF monotherapy.

## 2 Case description

### 2.1 Case 1 (poor response to anti-VEGF monotherapy)

**Figure 1:**
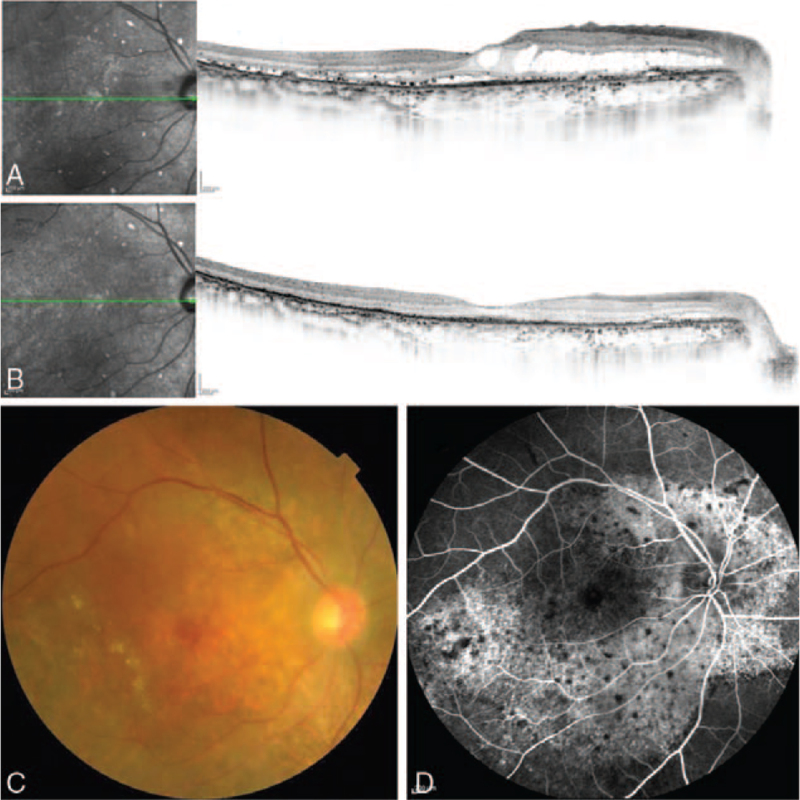
Case 1: Images of the right eye. Spectral-domain optical coherence images acquired 1 week before (A) and 7 months after (B) the combined therapy. Intraretinal fluid and subretinal fluid appears resolved, while the dry status is maintained. However, the outer retina is damaged. A color fundus photograph acquired 1 week before the therapy (C). Multiple red patches and hard exudate is observed. An early-phase fluorescein fundus angiogram acquired 1 week before the therapy (D). Diffuse leakage along the arcade vessels and multiple block signs are observed.

A man in his 70s on prednisolone, mesalazine, and ciclosporin for hypoplastic anemia and ulcerous colitis, visited a nearby hospital for active vision loss in both eyes. On examination, hard exudate, intraretinal fluid (IRF), and subretinal fluid (SRF) were observed. One triamcinolone acetonide subTenon injection (TASTI) and subsequent 3 to 4 intravitreal 0.5 mg ranibizumab injections (IVR, Lucentis; Novartis, Buläch, Switzerland) were administered in both eyes; however, the exudate persisted. Therefore, 16 months after onset, he was referred to our hospital. Upon presentation, his best-corrected visual acuity (BCVA) was 20/40 and 20/50 in the right and left eye, respectively. Characteristic multiple red patches and exudate were observed in both eyes (Fig. 1). Retinal specialists diagnosed the case as BDUMP based on the characteristic fundus findings. Combined IVR and normal-fluent PDT (Visulas PDT system 690S; Carl Zeiss) intravenous verteporfin (Visudyne; Novartis, Basel, Switzerland) administration (6 mg/patient's body surface area [m^2^]) were performed for both eyes because this treatment was expected to more strongly suppress retinal exudate than anti-VEGF monotherapy and TASTI. The exudate resolved 1 to 4 weeks after treatment initiation. No additional treatment was required thereafter at the final visit, and 7 months after the therapy, his BCVA was 20/30 and 20/50 in the right and left eye, respectively. The combination therapy helped effectively manage the retinal exudate, which did not respond to anti-VEGF monotherapy and TASTI. However, posterior subcapsular cataract progressed rapidly, which necessitated cataract surgery. At the final visit, 10 months after the therapy, no recurrence of exudate was observed, and his BCVA was 20/30 and 20/20 in the right and left eye, respectively. No adverse event occurred in the retina after the combination therapy.

### 2.2 Case 2 (poor response to anti-VEGF monotherapy)

**Figure 2:**
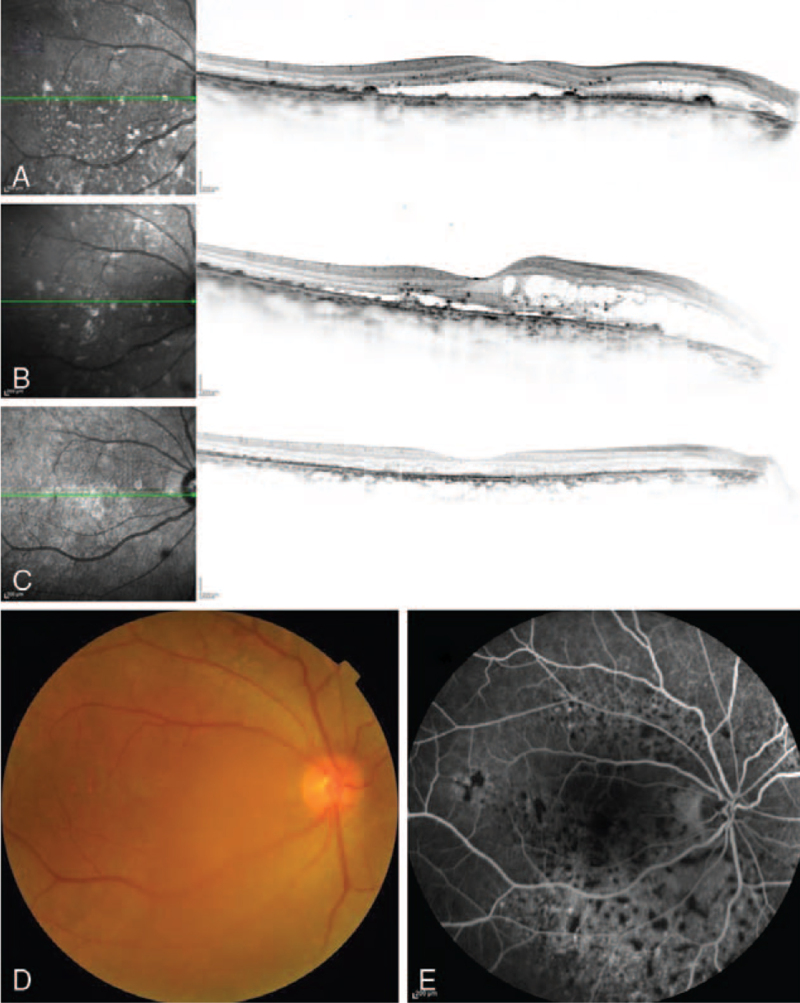
Case 2: Images of the right eye. Spectral-domain optical coherence images acquired 15 (A) and 5 months before (B) and 2 years after (C) the combined therapy. The outer retinal damage progressed and the intra retinal fluid increased during the 10 months before the therapy (A, B). Although intraretinal fluid and subretinal fluid resolved, the dry status persisted after the therapy, and outer retinal damage is seen (C). Exudate occurred earlier in the right eye than in the left. Despite exudate resolution, best-corrected visual acuity of the right eye was low because of the outer retinal damage. A color fundus photograph acquired 5 months before the combined therapy (D). The image is slightly blurred because of the posterior subcapsular cataract. Multiple red patches and hard exudate are observed. An early-phase fluorescein fundus angiogram acquired 5 months before the therapy (E). Diffuse leakage along the arcade vessels and multiple block signs are observed.

A man in his 60s, on prednisolone therapy for adult Still disease—a rare systemic autoinflammatory disease—visited a nearby hospital with active vision loss in the right eye. Hard exudate, IRF, and SRF were observed. Monthly intravitreal 2 mg aflibercept injections (IVA, Eylea; Bayer, Basel, Switzerland) were administered twice for the right eye; however, the exudate persisted. Therefore, he was referred to our hospital 5 months after symptom onset. Characteristic multiple red patches and exudate were observed in both eyes and his BCVA was 20/22 and 20/17 in the right and left eye, respectively (Fig. 2). Eventually, retinal specialists diagnosed the case as BDUMP based on the characteristic fundus findings. As the posterior subcapsular cataract rapidly progressed during the 1-year observation, bilateral cataract surgery was performed. However, postoperatively, the exudate persisted and his BCVA was 20/200 and 20/33 in the right and left eye, respectively. Combination therapies were performed for both eyes 2 years after the onset, similar to patient 1. The exudate eventually resolved 1 to 3 months after the initiation of therapy. No additional treatment was required for 2 years post-therapy. At the final visit, 2 years after the therapy, no recurrence of exudate was observed, and his BCVA was 20/200 and 20/22 in the right and left eye, respectively. No adverse event occurred after the combination therapy. The combination therapy effectively managed the retinal exudate, which did not respond to anti-VEGF monotherapy.

### 2.3 Case 3 (relatively good response to anti-VEGF monotherapy)

A man in his 70s, on prednisolone therapy for polymyalgia rheumatica, giant cell arteritis, and pancreatic body tumor, visited a nearby hospital with blurred vision. Hard exudate, IRF, and SRF were observed. One IVA was administered for the left eye 5 months after symptom onset. Although IRF disappeared, residual SRF were observed. Because of persistent bilateral blurred vision and because he had a history of cerebral infarction, 1 triamcinolone acetonide sub-Tenon injection was administered for the both eyes 1 month after the IVA. Blurred vision and IRF progressed. Therefore, he was referred to our hospital 7 months after the onset. Multiple red patches and exudate were observed in both eyes and his BCVA was 20/50 and 20/30 in the right and left eye, respectively. Eventually, retinal specialists diagnosed the case as BDUMP based on the characteristic fundus findings. Three monthly IVA were administered for both eyes 9 months after the onset. The exudate resolved 2 months after the therapy. At the final visit, 4 months after the therapy, no recurrence of exudates was observed, and his BCVA was 20/66 and 20/100 in the right and left eye, respectively. He was lost to follow up. No adverse event occurred after the therapy at the time of his last visit.

## 3 Discussion

This study described 2 cases of BDUMP, an extremely rare disease, which were treated with combined IVR and PDT after failing anti-VEGF monotherapy and/or TASTI treatments. Posttreatment, the exudate did not recur during the 7-month and 2-year follow-up periods. Another case of BDUMP was treated with 3 rounds of monthly anti-VEGF monotherapy doses and showed no recurrence for at least 4 months.

Several treatment methods for BDUMP have been reported. Intravitreal anti-VEGF monotherapy, systemic carbonic anhydrase inhibitor, intraocular methotrexate, and intravenous immunoglobulin did not improve BCVA or serous retinal detachment.^[2]^ In the present study, anti-VEGF monotherapy was effective for at least 4 months in patient 3. It is possible that other patients would have no requirement of additional treatment after anti-VEGF monotherapy in BDUMP at constant rate. A previous reported that periocular injection of triamcinolone acetonide 40 mg reduced subretinal fluid for 5 months and was effective for recurrence.^[3]^ In the present study, patient 1 underwent TASTI; however, it was not effective for retinal exudate. Our findings potentially propose that anti-VEGF monotherapy is first administered for BDUMP and combination therapy of PDT and IVR is administered for cases with poor effectiveness of the anti-VEGF monotherapy as a rescue treatment.

Herein, lower pretreatment BCVA was associated with lower posttreatment BCVA. In patient 2, exudate occurred earlier in the right eye than in the left. Despite exudate resolution, BCVA of the right eye was low (20/200) compared with that of the left eye (20/22) because of outer retinal damage accompanied with long-term exudate. Thus, early diagnosis and prompt treatment including the combination therapy for resolution of the retinal exudate should prevent BCVA decreases.

The diagnosis of BDUMP is based on the characteristic fundus findings in accordance with Gass criteria^[1]^; however, it is sometimes difficult. BDUMP is similar to neovascular age-related macular degeneration and central serous chorioretinopathy (CSC).^[6,7]^ In this report, all patients had exudative retinal detachment and multiple, round or oval, sable, red patches at the level of the RPE in the posterior fundus. They had no choroidal neovascularization. Among 3034 patients who visited Kyoto University Hospital Macular Service between February 2014 and September 2020, only 3 patients (0.1%) had the characteristic findings with physical disorders. However, 2 cases did not have classic cancers-associated with BDUMP. There remains a possibility of CSC secondary to steroids. The effectiveness of PDT for CSC has been reported.^[8]^

In conclusion, combined IVR and PDT may be a feasible therapeutic option for treatment-resistant exudate in patients with BDUMP. Early diagnosis of BDUMP and prompt administration of combination therapy are crucial.

## Author contributions

**Conceptualization:** Manabu Miyata.

**Data curation:** Manabu Miyata, Sotaro Ooto, Masayuki Hata, Ayako Takahashi, Akitaka Tsujikawa.

**Funding acquisition:** Manabu Miyata.

**Investigation:** Manabu Miyata, Sotaro Ooto, Masayuki Hata, Ayako Takahashi, Akitaka Tsujikawa.

**Methodology:** Manabu Miyata.

**Writing – original draft:** Manabu Miyata, Sotaro Ooto, Masayuki Hata, Ayako Takahashi, Akitaka Tsujikawa.

**Writing – review & editing:** Manabu Miyata.
